# Synchrotron radiation imaging analysis of neural damage in mouse soleus muscle

**DOI:** 10.1038/s41598-020-61599-7

**Published:** 2020-03-12

**Authors:** Jiwon Lee, Sang-Hun Jang, Suk-Jun Lee, Onseok Lee

**Affiliations:** 1 Department of Computer & Science Engineering, Graduate School, Soonchunhyang University, 22, Soonchunhyang-ro, Asan City, Chungnam 31538 Republic of Korea; 2 Department of Physical Therapy, College of Health and Life Science, Korea National University of Transportation, 61, Daehak-ro, Yonggang-ri, Jeungpyeong-eup, Jeungpyeong-gun, Chungcheongbuk-do 27909 Republic of Korea; 3 Department of Biomedical Laboratory Science, College of Health & Medical Sciences, Cheongju University, 298, Daesung-ro, Cheongju City, Chungbuk 28503 Republic of Korea; 4 Department of Medical IT Engineering, College of Medical Sciences, Soonchunhyang University, 22, Soonchunhyang-ro, Asan City, Chungnam 31538 Republic of Korea

**Keywords:** Three-dimensional imaging, Preclinical research

## Abstract

Damage to lower limb muscles requires accurate analysis of the muscular condition via objective microscopic diagnosis. However, microscopic tissue analysis may cause deformation of the tissue structure due to injury induced by external factors during tissue sectioning. To substantiate these muscle injuries, we used synchrotron X-ray imaging technology to project extremely small objects, provide three-dimensional microstructural analysis as extracted samples. In this study, we used mice as experimental animals to create soleus muscle models with various nerve injuries. We morphologically analyzed and quantified the damaged Section and Crush muscles, respectively, via three-dimensional visualization using synchrotron radiation X-ray imaging to diagnose muscle injury. Results of this study can also be used as basic data in the medical imaging field.

## Introduction

Muscles maintain physical balance in daily life, and are essential for preventing physical disability. In particular, muscles of the lower extremities play a very important role in maintaining posture through normal gait and weight support. Moreover, damage to the muscles of the lower limbs may lead to considerable difficulties in daily life because of their close interaction with other parts of the body, such as the arms and torso. In addition, lower-limb muscle damage is closely associated with degenerative conditions, such as arthritis and osteoporosis^1^.

The sciatic nerve begins from the sacral plexus and extends along the tensor fasciae latae muscle and through the calf to the foot^2,3^. The sciatic nerve is the largest nerve in the human body, and is divided into multiple peripheral nerves that innervate the sensory and motor areas of the lower limbs. Injury to the sciatic nerve can trigger muscle fatigue, aging, atrophy, and lower-limb dysfunction^4–11^. Among the muscles dominated by the sciatic nerve, the soleus muscles exhibit significant regenerative capacity to promote functional recovery^8–11^. Furthermore, when muscle atrophy is prominent, less myogenin is expressed to increase elasticity against muscle damage^12^.

Studies investigating these phenomena usually conduct histochemical analyses using animal experiments^8,9,11^. Microscopic imaging and micro-CT imaging have also been employed to observe changes (such as injury or recovery) observed from the condition of mouse soleus muscle. Existing studies on imaging of this subject have been performed by identifying changes in the muscle shape due to hypertrophy in mice^13^, determining the effects of treatment on muscle atrophy^14^, and measuring the effects on sciatic neuralgia^15^ using microscope or micro-CT.

Microscopic imaging yields high-resolution images (up to nm levels) depending on the equipment^16^. In the clinic, to achieve the highest accuracy, a biopsy is obtained to establish the diagnosis^17,18^. Thus, imaging is indispensable for histological analysis. The nomenclature has been standardized in a previous study via histological analysis of lesions involving soft tissue, skeletal muscle, and mesothelium in mice and rats using an electron microscope^4^. Other studies have histologically analyzed the microscopic changes in muscle regeneration following leucine supplementation, which induced the recovery of skeletal muscle in older mice^5^. Transmission electron microscopy facilitates cross sectional tissue analysis from its excellent discrimination; however, it provides two-dimensional cross sections for limited sites of a sample. Three-dimensional image reconstruction is a time-consuming task because of complex processing and procedures requiring slide images to be stacked. Scanning electron microscopy yields stereoscopic images of the sample surface and enables visualization of the 3D surface by reconstruction. Other techniques are often used to facilitate 3D reconstruction or quantitative evaluation. For example, micro-CT imaging is mainly used in small animal research, and elucidation of internal structures is based on X-ray analysis. It also yields high-spatial-resolution of images in a short time and 3D sample modeling via reconstruction. Therefore, quantitative evaluation is feasible and represents an excellent imaging technology. Depending on parameters including equipment and slice thickness, it can yield a resolution of up to 10 µm^19,20^.

Here, we describe X-ray imaging using synchrotron radiation with special characteristics compared with micro-CT imaging. Synchrotron-based X-ray imaging yields images with high spatial resolution ranging from several micrometers (approximately 5 µm) to tens of nanometers (approximately 30 nm) depending on the wavelength and devices^21–24^. The synchrotron light source exhibits excellent focusing properties through partial coherence based on very short wavelengths $({10}^{-7}\, \sim \,{10}^{-10}$ m) and diffraction. The monochromatic light selected here exhibits excellent focusing characteristics through coherence-based interference and contributes to greater resolution. Therefore, it is possible to obtain high-resolution images of very small objects and to image internal microstructure of materials. By reconstruction through the nature of X-ray imaging, the 3D surface shape, microstructure, sagittal, coronal, and axial cross sections are simultaneously displayed as three axes. Therefore, the internal structure is freely analyzed in various axial directions. In addition, sample preparation can be simplified because the tissue-sectioning process is omitted^7,25–33^. Because the superior spatial resolution facilitate 3D modeling and quantitative evaluation of microscale bio-samples, it can have shown enhanced identification of structures that are difficult to observe in conventional imaging or micro-CT imaging^33^. Studies have been conducted using human breast tissue, nails, and hair, and rodent tissues, bones, blood vessels, and muscles using this technology^7,25–33^.

We objectively assessed the extent of muscle tissue damage or recovery using synchrotron radiation. The soleus muscle with an injured sciatic nerve can be modeled by either crushing the nerve or sectioning it. In the Crush model, the muscles atrophied over time and gradually recovered. In the Section model, the muscle fibers continued to undergo muscular atrophy over time and the fibers changed from polygonal to round. The normal soleus muscle without sciatic nerve injury showed muscle development, as the size of the muscle fibers increases with growth^34^. Our study is based on visualization of nerve injury in muscle, and presents a new 3D diagnostic analysis. In addition, animal models of various diseases, such as atrophy, degeneration, edema, fibrosis, inflammation, and necrosis, facilitate the development of diagnostic criteria for objective visualization and comparative analysis.

In this study, we propose a novel method of objective image analysis for muscle diagnosis using synchrotron radiation imaging and quantification of nerve injuries in mouse muscle. The purpose of this, however, is not to quantify the degree of nerve injury in the muscle by gathering data based on multiple samples. Synchrotron radiation imaging can be used to acquire several hundreds of tomography images in each sample simultaneously, including the internal structure of the entire sample. Therefore, this study aims to quantify muscle nerve injury in a single animal sample. This may complement the difficulties or limitations existing in conventional histology and micro-CT imaging. This study attempts to confirm the microstructure and morphological changes in mouse muscle fibers. Accordingly, we attempted to visualize and quantify the degree of nerve injury of muscle fibers in nerve-damaged muscle models of a single mouse sample.

## Results

### Muscle weight

After injury to the sciatic nerve, muscle weights were measured to confirm the atrophy of the soleus muscle. The sample weights were measured before ethanol dehydration and immediately after dehydration, but before staining. The samples of the soleus muscle with injured sciatic nerve are in the Injury, and those of the soleus muscle with intact nerve are in the Non-injury. Four groups of muscle models were developed from mouse samples grown for four weeks at one-week intervals. The muscle weight increased slightly while the mice were growing and was measured with a microelectronic scale to 4 decimal places for simple comparison. The liquid reagent on the sample was dried very carefully using experimental wipers.

**Figure 1:**
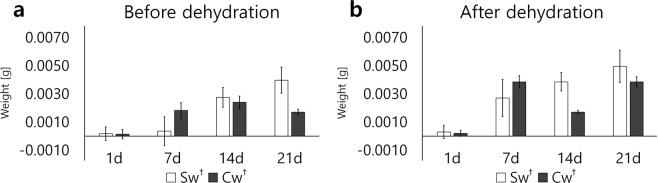
The degree of nerve injury for mouse soleus muscles was represented at 1-week intervals. The injury models, relative to each non-injury model, represent the extent of injury as the length of the bar graph. To confirm muscle injury, the muscle weight was measured before and after dehydration during sample preparation. The section models show an increase in tissue injury gradually (Sw), and the crush models show a decrease in injury after a certain period (Cw). Each data was significant for P-value 0.05.

To determine the change in, that is, the degree of nerve injury, in the soleus muscle of the sciatic-nerve injury model, we subtracted the corresponding results in each Injury group from those in the Non-injury group. For example, in one mouse, the sciatic nerve of the left leg was sectioned, but not the right leg. The soleus muscles of the left leg represent the Section models in the Injury group, and the soleus muscles of the right leg are the Section models in the Non-injury group. To determine the extent of the injuries involving the soleus muscle in the left leg, the weight of the left leg was subtracted from that of the right leg, as shown in Fig. 1. The graphical bar shows the degree of injury in each model group. The extent of the injury represents that in the case of the Section groups increase, and in the Crush groups increase and decrease with the recovery following over time.

### Muscle shape image using microscope

**Figure 2:**
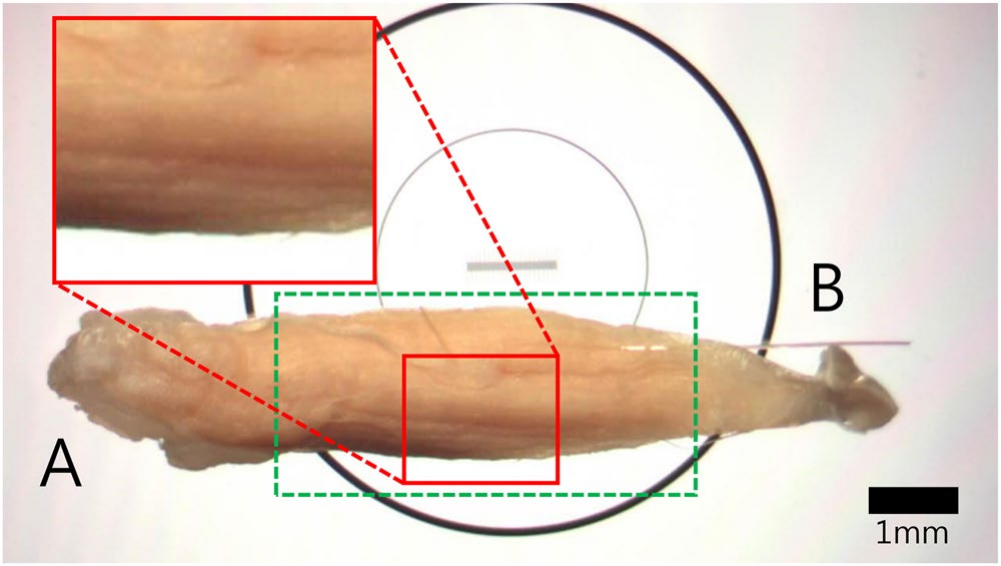
Microscope images of mouse soleus muscle. The 10× magnification shows a 21-day sample Section from Non-injury group. A represents the direction in which the tensor fasciae latae is located and B is the direction in which the Achilles tendon is located. The scale bar is 1 mm.

The soleus muscle in the Section for Non-injury model on day 21 after phosphotungstic acid (PTA) staining was imaged using a trinocular stereo microscope, model SG26T-PI (Daemyung, China/OEM) (Fig. 2). A MUC-500 camera (Wakayama, Chengdu, China) was installed to capture the images via a dedicated VMS program on a computer. Synchrotron radiation imaging was performed by positioning the field of view (FOV) at the belly of the muscle sample in a green box (Fig. 2). The enlarged image of the red box confirms the grain and curve in the surface of the muscle bundle based on the light reflected from the sample surface. The degree of injury to the external and internal structures of the muscle cannot be established via visual examination of the microscopic images.

### Analysis of histological microscopic imaging

Histological analysis was performed to identify the morphological changes in the mouse nerve-injury muscle models. The light microscope was an Olympus BX53 model (Olympus, Shinjuku, Japan), which yielded 400× magnification images with MetaVue Research Imaging Software (Molecular Devices, San Jose, CA, USA).

**Figure 3:**
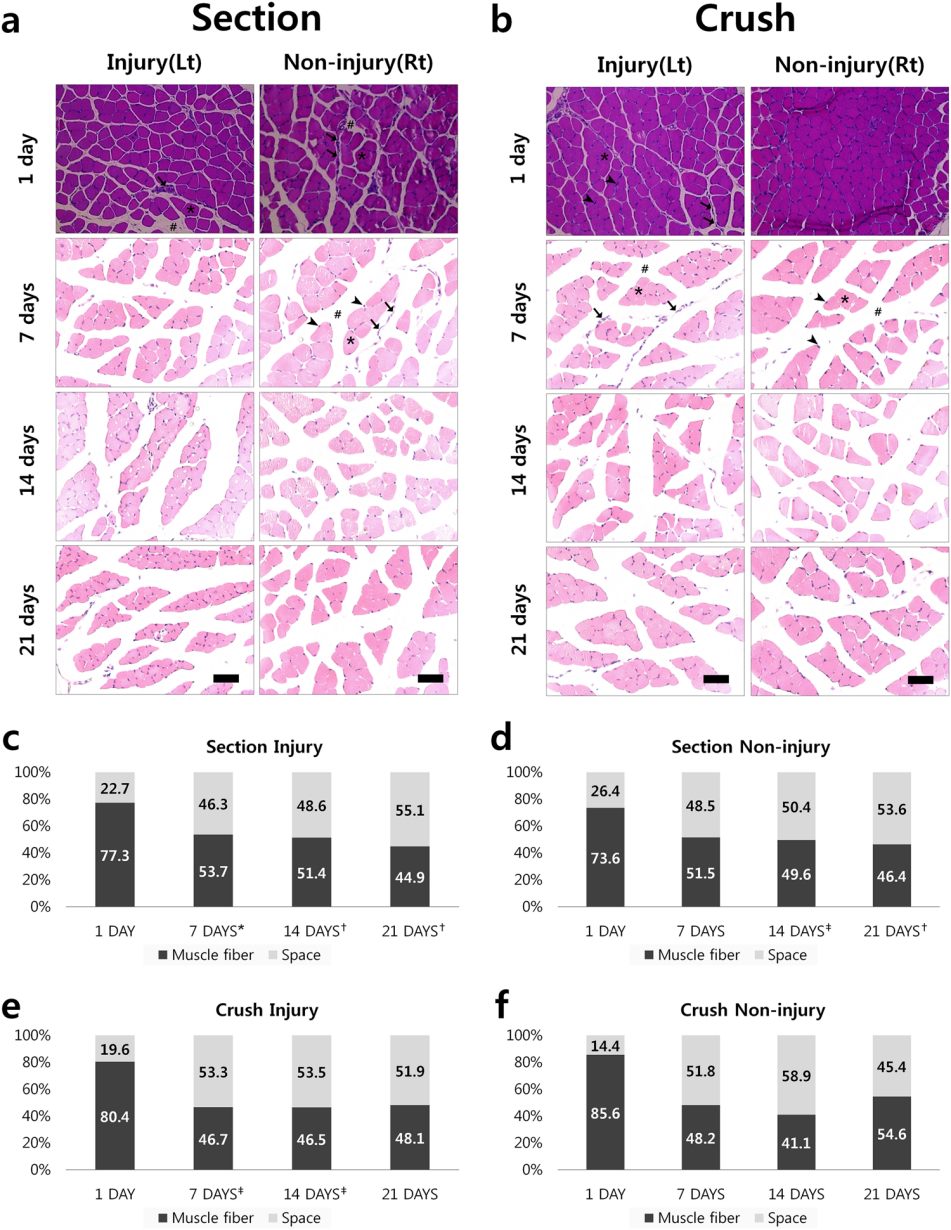
(**a**,**b**) Various models of nerve injury involving the mouse soleus muscle. Histological images representative of each model are shown confirming the cross-section of the internal structure of the muscle and changes according to the time difference after the injury. Arrowhead represents the nucleus, the arrow denotes fascia, the star is muscle fibers, and the hashtag refers to muscle fiber space. The scale bar is 25 μm. **(c**–**f**): A graph showing the ratio of fiber area to the injury area of the mouse soleus muscle in the histologic micrograph. Independent sample t-tests were performed to determine whether these morphological rates of change varied from 1-day images of H & E stained model in Fig. 3a,b had statistical significance. *p < 0.1, ^†^p < 0.05, ^‡^p < 0.01.

Images of the mouse soleus muscle were acquired after hematoxylin and eosin (H&E) staining and were analyzed based on the chronological sequence of injuries in each model (Fig. 3a,b). The muscle fiber space in the image represents a region showing the degree of muscle tissue injury. It contains adipose tissue, inflammatory cells, edema, and other hallmarks of tissue injury. The width of the space increased with the degree of injury^35–37^. In most of the tissue images, the fascia surrounding the muscle bundle were shed following atrophy and degeneration of intercellular cohesion. This space was enlarged under severe muscular atrophy. The muscle fibers of the Section model continued to atrophy with time. The shape of the fiber also changed from polygonal to round. The size of the muscle fiber with the smallest cross-sectional area was that of the Section sample on day 21. Muscular atrophy was the most prominent, following induction of muscle fiber space. In the case of the Crush model, muscles atrophied with time and gradually recovered after a specific period of time. The muscle fiber space increased for 14 days. The injury seemed to slow down after 21 days, similar to that after 14 days. The reduction was visually confirmed after injury. In the absence of injury, the Crush and Section models in the Non-injury group without direct nerve injury revealed a broad area of muscle fiber and muscle development. However, visual analysis of the cross-sectional images in a limited position cannot clearly establish the degree of damage overall damage to the soleus muscle sample.

Thus, for quantitative examination of identified features, we used ImageJ (Wayne Rasband, National Institutes of Health, USA) to measure the area of the soleus muscle fiber in the histological models (Fig. 3c–f). The area of the pixel-based muscle fibers was measured and expressed as a percentage. The area of the injury included the remainder of the region without muscle fibers. Other samples with time differences after injury based on day 1 of each model showed morphological changes. Statistical analysis confirmed significant difference in most of the morphological changes. In the Section model, the percentage of muscle fiber area decreased from 77.3% to 44.9% over time post-injury (Fig. 3c), and from 73.6% to 46.4% in the Section Non-injury model (Fig. 3d), suggesting that the injury was continuous in the muscle and that nerve injury on one side leg affects both leg muscles. The percentage area of muscle fiber space increased from 22.7% to 55.1% and from 26.4% to 53.6%, respectively (Fig. 3c,d). Because the fascia surrounding the muscle fibers were damaged by injury, the muscle fiber space expanded gradually and the degree of injury worsened. Based on these results, the muscle injury was identified objectively. In the Crush models, a rapid change in the ratio of muscle fiber to muscle fiber space area was confirmed after a certain period (Fig. 3e,f). Subsequently, the reduction in muscle injury again indicated recovery of the muscle tissue. However, the results of this image analysis were obtained using 2D plane images. It is difficult to confirm the volume of injury in the soleus muscle and determine the overall height of the sample. A 3D reconstruction of microscopic images was obtained from slides at a specific site. This method is time-consuming, and it is difficult to stack slide images. Analysis of the 3D volume is necessary to determine the extent of injury more accurately in a single model. Therefore, we performed X-ray imaging to project the entire internal muscle structure.

### Three-dimensional visualization of mouse soleus muscles

**Figure 4:**
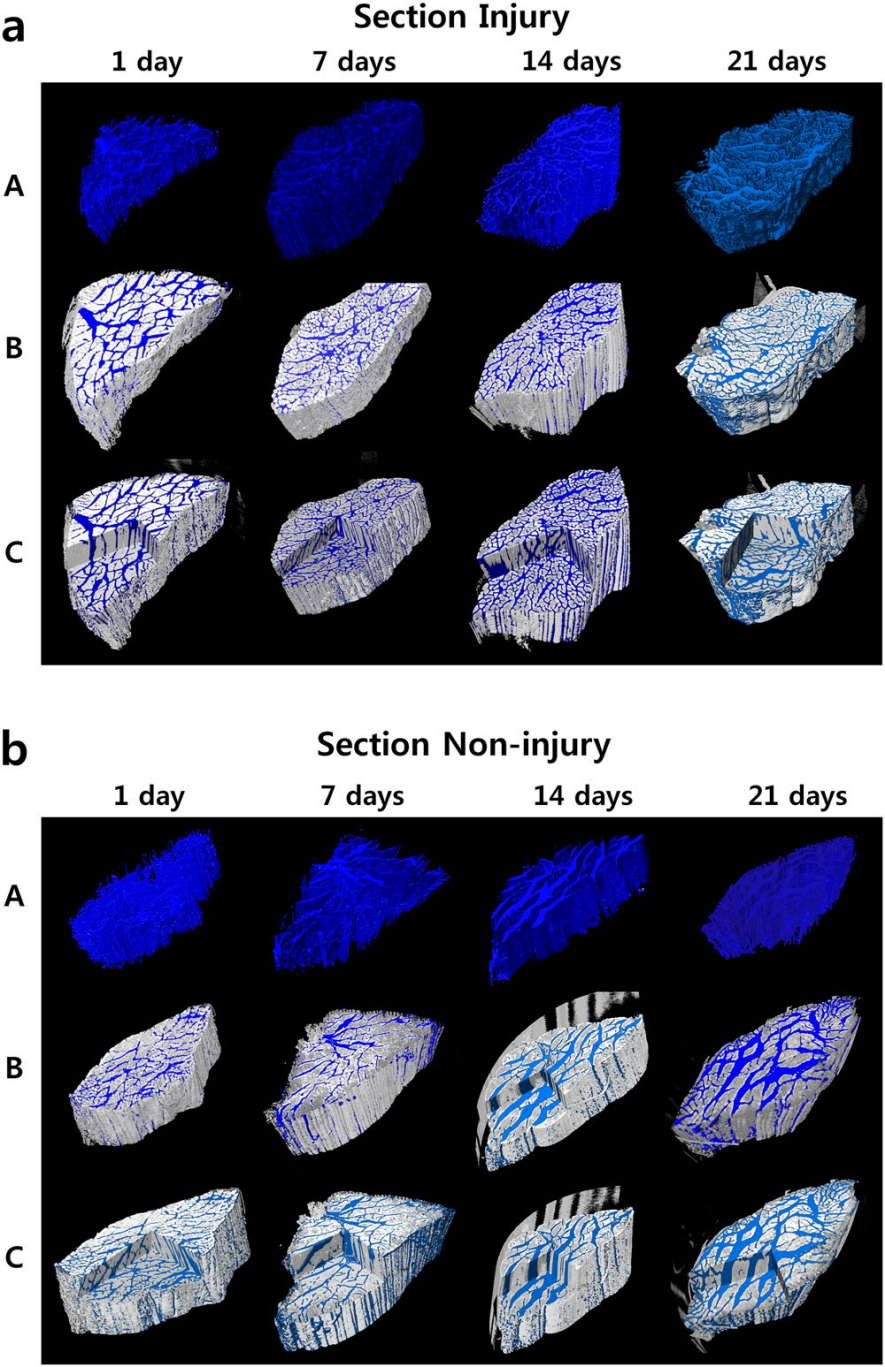
3D reconstructed and volume-rendered images of the soleus muscle on days 1, 7, 14, and 21 following nerve injury (**a**) and non-injury (**b**) in Section models. White area denotes muscle fibers. Blue area indicates muscle fiber space. Changes in space depend on the degree of injury. Row A with blue segments, and row B data are superimposed from row A on the 3D-reconstructed origin data. Here, line C shows morphological shape and internal structure of the sample at the same time. The Section model showed prominent injury with increased time after the injury.

**Figure 5:**
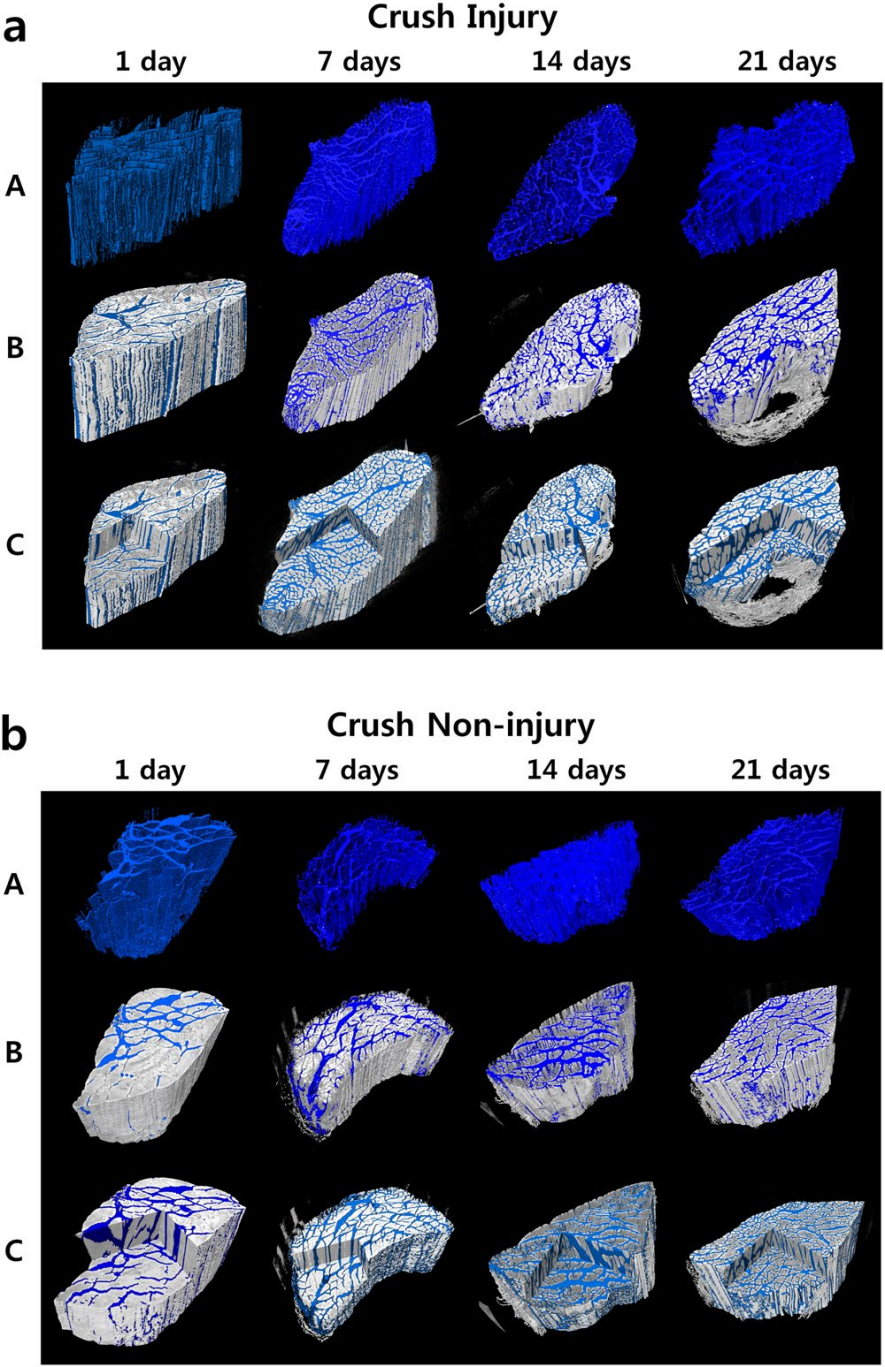
3D reconstructed and volume-rendered images of the soleus muscle on days 1, 7, 14, and 21 following nerve injury (**a**) and non-injury (**b**) in Crush models. White area denotes muscle fibers. Blue area is the muscle fiber space. Changes in space depend on the degree of injury. Row A with blue segments, and row B superimposes the data of row A on the 3D reconstructed origin data. Here, line C shows morphological shape and internal structure of the sample at the same time. The Crush model showed a gradual recovery after a specific period of time post-injury.

Three-dimensional volume-rendered images using phase-contrast tomography of the Section- and Crush-model mouse soleus muscles are presented in Figs. 4 and 5, respectively. The degree of nerve injury, as visualized in different samples, is displayed as 3D images, in which rows B and C reveal the overall form of the muscle and the white area represents muscle fibers. The blue area denotes muscle fiber space, showing the volume and the degree of muscle injury. Based on the severity of injury to the soleus muscle, it can be qualitatively distinguished from the prominently distributed volume of muscle fiber space. The greater the atrophy, the higher the degree of atrophy of the muscle fibers.

Row A represents a segmented and visualized image of the targeted muscle fiber space (Figs. 4 and 5). Studies representing the ambiguous area of the degree of injury in 3D are rare. Mouse muscles and the degree of injury were expressed in 3D. Their specific internal structures were morphologically segmented and quantitatively analyzed^38^. Synchrotron radiation imaging provides higher resolution and higher contrast images. Further, it can provide images of the soft tissue of small mouse muscles with a total sample length or height of less than 1 cm. In addition, it differs from the existing tissue-imaging methods, as it can be used to determine the microinternal structure by projecting the sample as-is without sectioning process.

Row B represents a superposition of the 3D muscle images of the original data on row A to confirm the location of the injury in the muscle (Figs. 4 and 5). The workstation and commercial program used to obtain these images facilitated simultaneous analysis of the axial, sagittal, and coronal planes of the samples, as well as image rendering. Line C shows this image representation. The volume of nerve injuries, in blue, was segmented using image-processing software. Compared with conventional microscopic images, the pattern and direction of the injury can also be observed in 3D, as the entire sample cross section can be confirmed in multiple locations. The degree of the injury and pattern of recovery for muscles are difficult to distinguish in these images, but can be quantified.

### Quantitative analysis and interpretation for images results

**Figure 6:**
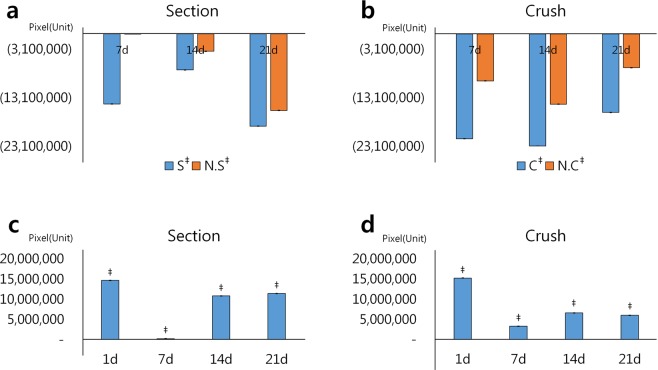
Graphs showing quantitative analysis of the volume of muscle fiber space segmented in 3D volume-rendered image. (**a**,**b**) Each sample is confirmed for progressive injury based on 1-day measurement. The length of the bar graph can confirm the extent or the pattern of recovery. In the figure, Crush (C), Section (S), and each Non-injury (N.C and N.S) model names are indicated by the first alphabets for brevity. (**c**,**d**) Data on the x-axis represent the degree of injury due to contralateral effect by measuring the difference between the Injury model and the corresponding Non-injury model. Graphs on both sides represent Section models with sustained injury, and Crush models with recovery patterns after a specific period. Independent sample t-tests were performed to determine statistically significant differences in the degree of injury between groups. ^‡^p < 0.01.

The 3D images acquired by synchrotron radiation imaging were composed of 300 seriate reconstructed images, including the belly region across the whole length of the soleus muscle samples. We were interested in the change in volume of muscle fiber space, where the nerve injury arises, and the muscle fibers following muscle injury in mice. Figure 6 shows the volume of muscle fiber space segmented in blue (Figs. 4 and 5). The expressed volume is the sum of the number of pixels at the segmented regions per slice, based on changes in the muscle fiber space according to the degree of injury. The standard deviation reveals the degree of injury segmented per slice. The figures represent progressive injury for each sample based on day 1 (Fig. 6a,b). The graph expressing values below zero may appear nearly negative. That is, damage must have occurred to the soleus muscle. However, the muscle samples differ in survival time at 1-week intervals, and thus, the injury in the surviving samples over 21 days showed a greater change compared with those 1 day after the nerve injury. Therefore, as the length of the bar graph decreases, the gradual increase in the muscle fiber space in the Section model represents severe injury. The Crush model showed eventual recovery. Thus, it is possible to analyze the degree of damage quantitatively in various muscle models of nerve injury.

In each sample, the difference between the Injury model and the corresponding Non-injury model was measured to confirm the degree of contralateral injury (Fig. 6c,d). The contralateral effect occurred on the opposite side from that left intact via neuropathic pain after peripheral nerve injury on one side of the same pain model^39–41^. In this study, the sciatic nerve, one of the peripheral nerves of mice, was used. The right legs were not injured; only the left legs were subjected to surgical injury. Two models were developed from the same mouse sample. Two types of nerve injury were induced surgically. Thus, a total of four models were constructed to confirm the same contralateral effect in both muscles of the same mouse. Statistical analysis via independent t-test showed significant results in all corresponding models. The graphs quantitatively confirm the degree of injury between the left and right sides of the mouse soleus muscle (Fig. 6c,d). Although only one day elapsed after the injury, the difference in the degree of injury between left and right sides was high because of the large changes on the side subjected to injury. Seven days after injury, both models showed the severity of atrophy. Thus, the difference in the degree of injury was low (Fig. 6c). In the Section model, however, injury to the left leg 14 days after surgical injury sharply increased, and was worse 21 days after injury. The degree of injury markedly increased compared with the uninjured side. In the case of the Crush model, the damage in the injured side was also more severe than in the non-injured side (Fig. 6d). The width of the injury also increased. After 14 days, the degree of injury between the legs in the model decreased.

## Discussion

This study investigated the morphological changes and quantitatively analyzed the degree of injury in mouse soleus muscles using synchrotron radiation imaging. Conventional microscopy of tissue cross sections yields excellent resolution. However, only limited and partial cross sections are visible, which prevents reconstruction of the overall 3D structure. In small animal studies, micro-CT may be used. Micro-CT and synchrotron radiation imaging use X-rays highly effectively. High-contrast internal imaging is facilitated by a relatively simple sample procedure, and 3D reconstruction and quantitative evaluation. However, the synchrotron radiation technology yields higher resolution than micro-CT, and the X-ray wavelength band of monochromatic light can be selected according to user needs, even with the same X-ray synchrotron source. Using these radiological techniques, animal experiments were conducted to obtain mouse soleus muscles for various types of nerve injuries, and 3D quantitative image analysis was performed to confirm the muscle condition and degree of injury in muscular diagnosis.

Finally, the 3D images obtained in this study were segmented into muscle fiber spaces to confirm the degree of atrophy of the soleus models with nerve injury. The degree of injury in each model was similar to that reported in previous studies. We also objectively analyzed the degree of muscle injury based on 3D data. In addition, injury to the peripheral nerve was analyzed quantitatively to confirm the contralateral effect on the non-injured side.

The numerical data showed some inconsistencies compared with the results of previous studies. Damage induced by sample extraction is difficult to handle realistically. Ideally, the same object should be evaluated at all time periods. This study proposed a method to quantitatively and visually analyze the 3D morphology of muscle tissue injury weekly using mice as the experimental subjects. It is practically impossible to use all the same parameters in this study. We used only 30 g mouse samples to conduct experiments under similar conditions. Our investigators with anatomical expertise extracted only the soleus muscle. However, the size of the body part varied slightly for each object, and sample variation was inevitable.

The number of laboratory animals used to perform 3D quantification of the muscle injury may have been low. As described above, synchrotron radiation imaging allows 3D reconstruction of the sample to full height, i.e., the number of tomography images acquired from a single sample with one-time synchrotron X-ray imaging can reach several hundred to several thousand slices. Although it is likely to yield results close to the those from analyzing the deviation of data obtained from many objects, quantitative comparison with large image data derived from only a single object might be adequate, consistent with our approach. Therefore, our method can be utilized as a diagnostic tool that recognizes the single original object condition for precise diagnosis considering various factors. In addition, it is possible to comprehend the elaborate pattern features of the sample as a large number of image data can be acquired. Quantification of the extent of nerve injury based on data acquired from multiple objects is not our goal.

Based on these animal experiments, it is possible to develop an automated system to objectively diagnose the degree of nerve injury in muscles in standardized animal studies. Further medical research conducted using synchrotron radiation can promote the development of cutting-edge medical technology for global applications. In addition, a 3D quantitative diagnostic method for the degree of injury to muscles may have a ripple effect in various lesions or fields. Above all, it is possible to confirm the advantages of this method using experimental animals and establish its reliability. In addition, it can promote the continuous development of the latest medical reference and diagnostic systems for disease recovery and treatment to highlight complex disease mechanisms.

In addition, because our synchrotron radiation technique generates extremely small micro-scale internal structure images, it can contribute to research and development of innovative new drugs for the diagnosis and treatment of muscle diseases via quantitative and objective analytical techniques. For example, it can be used to provide basic data for the development of revolutionary medicines by investigating the injury mechanism of degenerative disorders such as osteoporosis and arthritis. Thus, microstructural analysis can be used to determine 3D factors on the order of several microscale units in various muscle tissue injuries to develop new criteria enabling the use of synchrotron radiation in related studies when there is a lack of analytical equipment. Basic research for the quantitative diagnostic analysis of nerve-injured muscle tissue can also be applied in medical diagnostic imaging of other diseases.

Furthermore, the synchrotron source can be used to diagnose biosamples of disease models; related technological studies continue to this day^42^. It has been mentioned that clinic feasibility is expected in specific medical imaging, such as breast CT imaging, through the use and development of synchrotron source-based compact light sources^43^. Although they may be difficult to use in clinical practice, owing to the practical limitations of synchrotron facilities, synchrotron images could provide a gold standard for evaluating image quality to understand the nature of samples^44^.

Finally, with regard to muscle contraction, there are still technical difficulties to identify molecular-scale biomaterials, such as the combination of myosin-binding protein and actin, and to understand the interaction of sarcomere structures. Because theories remain unproven regarding a physical mechanism related to sliding between actin and myosin filaments, which exist at intervals of approximately 45 nm in the mouse sample, electron micrographs have been used commonly to investigate this^45^. Therefore, we plan to research the possibility of confirming the existence of these structures in the future using high-resolution X-ray images with this synchrotron imaging method.

## Methods

### Ethics statement and experimental animals

Ethical approval was obtained from the Institutional Animal Care Use Committee of the Soonchunhyang University (SCH IACUC) of the Republic of Korea (approval number: SCH18-0021). All animal experiments were performed in accordance with the relevant named guidelines and regulations. The experimental animals used were eight-week-old ICR mice. Male mice weighing 30 g each were used to avoid the possibility of pregnancy.

Mice were classified into four groups of six (n = 24). The nerve controlling the soleus muscle differed in the mechanism of injury. The sciatic nerve was located laterally on the tensor fascia latae muscle was one of the peripheral nerves subjected to nerve injury. The soleus muscles in the left leg of the mouse in the Injury group were subjected to sciatic nerve injury. These were divided into two model groups: The model obtained by cutting the nerve was called Section, and the other, obtained by crushing the nerve for 15 s, was called Crush^34,46–49^. The soleus muscles of the right leg in the mouse with the intact nerve were assigned to the Non-injury groups. They were also categorized into the contralateral sides in the leg of the soleus muscle model in the Section and Crush models. Surgery was performed on the same day in all animals to injure the nerve. All the mice were sacrificed to acquire samples after surviving for 1, 7, 14, and 21 days after surgery. The one-week interval difference after injury was to determine the degree of muscle nerve injury, recovery, and developmental level in the experimental animals.

### Sample preparation

Sample staining was processed with PTA and hematoxylin and eosin. It is difficult to acquire sharp or clear X-ray images without staining the muscles of the soft tissues prior to imaging. This is because the X-ray absorption of the elements that compose the tissue is low. Therefore, PTA staining was used to enhance the contrast of tissues. Dehydration increased the reagent permeation into the tissue. The same experimental conditions showed the same artifacts. The PTA reagent (Quintiles, Surrey, UK) was prepared at a concentration of 0.3%^28^. H&E staining was performed to analyze the histological images by first treating the soleus muscles with formalin and washing sufficiently under flowing water, followed by dehydration from low-density ethanol to a high-density gradient to prevent twisting. After they were cleaned with Xylene, the samples were fixed with paraffin tissue and sectioned with a microtome into 4-μm-thick slices. The remaining paraffin was adhered to the slide and removed with xylene and ethanol. The slices were subjected to H & E staining using VENTANA HE 500 (Roche, USA).

### Synchrotron radiation

**Figure 7:**
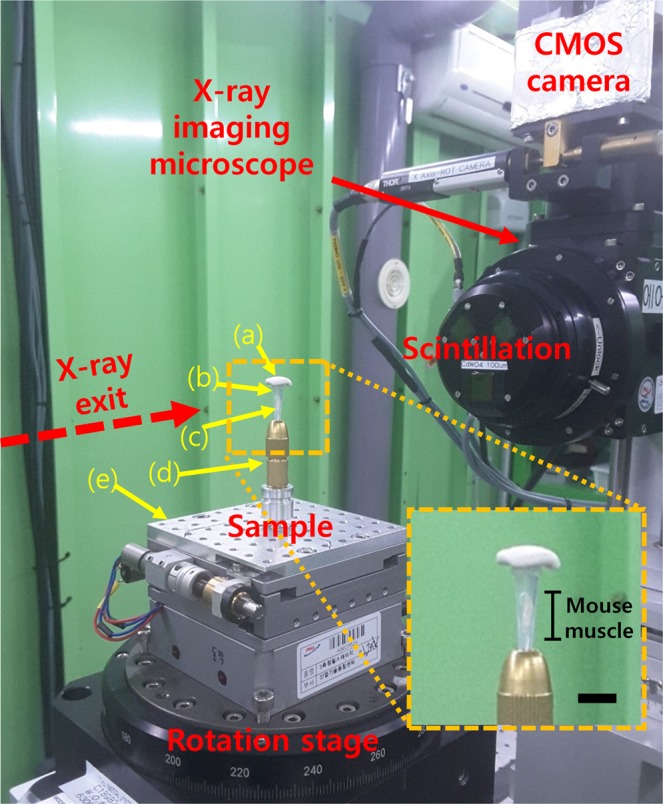
Phase-contrast hard X-ray microscopy of PLS 6C beamline. A monochromatic X-ray beam emitted along the red dotted line (**a**) was used to block the tube entrance with blu-tack, a fixing adhesion rubber. (**b**) Pipette tip (0.5 to 10 μl). (**c**) Soleus muscle sample of an experimental animal mouse. (**d**) Magnetic base provided by 6C BMI. (**e**) Stage rotating 360 degrees. The enlarged image reveals the sample used in this study located in the tube. The scale bar is 5 mm.

The experimental site in this study was the three-generation Pohang Light Source (PLS-II) of the Pohang Accelerator Laboratory (PAL) located in Pohang, South Korea. The synchrotron electron energy was 3.0 GeV and the experimental beamline was 6C Bio Medical Imaging (BMI) (Fig. 7). Absorption and phase-contrast X-ray imaging were performed in the 6C beamline. Using the multi-pole wiggler, monochromatic X-rays of 10 to 60 keV were generated with excellent transmission. In this experiment, synchrotron-based phase-contrast X-ray tomography imaging was performed, and a 30 keV X-ray beam was selected with wavelength tunability of monochromatic light.

The X-ray beam emitted in the direction of the arrow in the red line illuminated the sample positioned on the rotation stage (Fig. 7). The light transmitted through the sample revealed the details of the sample tissue. When the light was focused on the scintillator screen attached to the optical microscope, it was visualized according to the phase-contrast information of the light based on the internal structure of the sample. This image was magnified by a microscope objective lens (UPLSAPOO4X, Olympus, Japan), and the spatial distribution of the sample with respect to the X-ray intensity was recorded through a camera with an attached microscope. The X-ray imaging microscopes (Optic Peter, Lentilly, France) were equipped with CMOS cameras (Zyla Andor, Belfast, UK). The objective lens used 4× magnification. The resolution was 1.625 µm. The exposure time was 500 ms during the projection, and the distance between the microscope camera and the sample was 15 cm.

CT reconstruction was performed using the Octopus Program (Inside Matters, Aalst, Belgium)^50,51^. Symmetry around the axis of rotation could be expected in the best reconstruction. The AMIRA program was used for 3D visualization and analysis. Our performance process is as follows^21,52^.

1. In all steps, some areas were manually segmented and post-processed using the brush tool.
2. The median and non-local means filters were used for noise removal. The median filter is often used for pre-processing in image enhancement, such as edge detection^53^. The non-local means filter is characterized by preserving the edges using gradient information extracted from the image^54,55^. In this study, we tried to segment the muscle fibers and muscle fiber spaces to determine the degree of muscle injury. However, the boundaries of the structures were not clear, and there were areas where segmentation was difficult. Therefore, the combination of both improves the image boundary and removes the noise effectively.
3. The initial approach is to label the muscle fiber space with a seed point using the magic wand tool. Because this area is considered similar to the background intensity, an image-segmentation method based on region growing was selected^53^ and inverted, and then the muscle fiber was labeled.
4. Noise generated after segmentation was removed by applying island removal.
5. Next, we applied closing to the segmented muscle fiber, which preserves the shape and size of the object^53^, and performed hole filling to obtain the 3D contours of the muscle bundles^56^.
6. The muscle fiber space was obtained by subtracting the muscle fiber data obtained in step 3 from the contour 3D data. This was performed with the top-hat transform as a local thresholding tool.

### Statistical processing

The statistical program used in this study was IBM SPSS Statistics 21(IBM, New York, USA). We performed an independent-sample t-test to identify the degree of differences between groups for each muscle model within a specific time period after nerve injury and determined that it was significant.
